# Computational Prediction of the Epitopes of HA1 Protein of Influenza Viruses to its Neutralizing Antibodies

**DOI:** 10.3390/antib8010002

**Published:** 2018-12-20

**Authors:** Xiaoyan Zeng, Fiona S. Legge, Chao Huang, Xiao Zhang, Yongjun Jiao, Herbert R. Treutlein, Jun Zeng

**Affiliations:** 1 Institute of Pathogenic Microbiology, Jiangsu Provincial Center for Disease Prevention and Control, Key Laboratory of Enteric Pathogenic Microbiology, Ministry of Health, Nanjing 210009, China; zengxiaoyan6164@sina.com (X.Z.); nestar1987@126.com (C.H.); yongjunjiao@jscdc.cn (Y.J.); 2 MedChemSoft Solutions, Level 3, 2 Brandon Park Drive, Wheelers Hill, VIC 3150, Australia; suelegge@gmail.com; 3 Key Laboratory of Antibody Technique, Ministry Health, Nanjing 210029, China; zhangxiao@njmu.edu.cn; 4 Sanoosa Pty Ltd., Level 30, 35 Collins Street, Melbourne, VIC 3000, Australia; herbertin@gmail.com

**Keywords:** multiple copy simultaneous search, computational chemistry, antibodies, epitope mapping

## Abstract

In this work, we have used a new method to predict the epitopes of HA1 protein of influenza virus to several antibodies HC19, CR9114, BH151 and 4F5. While our results reproduced the binding epitopes of H3N2 or H5N1 for the neutralizing antibodies HC19, CR9114, and BH151 as revealed from the available crystal structures, additional epitopes for these antibodies were also suggested. Moreover, the predicted epitopes of H5N1 HA1 for the newly developed antibody 4F5 are located at the receptor binding domain, while previous study identified a region 76-WLLGNP-81 as the epitope. The possibility of antibody recognition of influenza virus via different mechanism by binding to different epitopes of an antigen is also discussed.

## 1. Introduction

Influenza virus has been a pandemic threat for human. In last century, three human influenza A pandemics (1918 H1N1 Spanish, 1957 H2N2 Asian, and 1968 H3N2 Hong Kong) have killed MILLIONS of people worldwide [1,2,3,4]. In the past few years, several infection and transmission in humans by the highly pathogenic H5N1 avian flu in southeast Asian countries have heightened fear that the next influenza pandemic is due [5,6,7].

Current therapeutics for influenza A viruses consists of two classes of drugs, the admantanes (amantadine and rimantadine) and the neuraminidase inhibitors (oseltamivir and zanamivir) [8]. However, the virus has developed drug resistance to these drugs [9,10,11]. Alternative strategies to combat the constant threats by influenza could be passive immune-prophylaxis with monoclonal antibodies (mAbs) which recognize broadly conserved influenza epitopes with broad range neutralizing activities [12,13]. The most important protective antigen on the surface of influenza virus is HA [14], a glycoprotein composed of HA1 and HA2 subunits. Three monomers of HA form a homo-trimeric protein that performs fusion of the viral and host membranes. HA1 functions as the receptor binding domain while HA2 acts as membrane fusion domain [14]. HA2 domain is rich with alpha-helices, which can form hydrophobic pockets and facilitate binding with the antibodies [15]. HA1 consists of three helices, but the antibodies against these sites with cross-reactivity to other viruses have not been previously reported [16].

While most neutralizing antibodies against the influenza HA recognize epitopes in the hyper-variable region that surrounds the receptor binding site and interferes with binding to host cells [17,18,19,20], other antibodies such as AbCR6261 and AbCR9114 were found to bind to the HA1 and HA2 proteins at their membrane fusion part so as to block the entry to host cell [21,22]. These antibodies were isolated from phage display selection on recombinant H5 HA, and can neutralize several influenza subtypes such as H1, H2, H5, H6, H8, and H9 [21]. Other antibodies such as AbBH151 and AbHC45 are found to bind at the vestigial esterase domain [18]. Recently, a human 4F5 single-chain Fv antibody has been developed from a library of phage-displayed human scFv generated from lymphocytes of H5N1 virus vaccinated individuals [23]. While a conserved epitope (76-WLLGNP-81) of HA1 domain was shown to neutralize the H5N1 virus, details of 4F5-H5N1 recognition is still unclear.

We have developed a new approach for predicting the antibody-binding epitope of an antigen [24]. This method was successfully applied to predict the epitopes of ecodomains of glycoproteins of a bunyavirus, “severe fever with thrombocytopenia syndrome (SFTS) virus”, to its human antibody Mab 4–5 [24], Shiga Toxin 2 (Stx2) subunit A to its specific antibodies 11E10 and S2C2 [25]. More recently, it has been used to identify the epitopes of Dengue Virus NS1 protein to its antibodies [26], and the epitopes of human papillomavirus 16 (HPV16) L1 proteins to its antibodies AE3 and AG7 [27]. It involves the location of minima of chemical functional groups on the key region of the antibody using an exhaustive “multiple copy simultaneous search” (MCSS) approach [25,28], identifying the cluster pattern of MCSS minima of a specific functional group which are subsequently converted into the amino acid sequence pattern on the surface of the antigen, and search the sequence pattern over the antigen protein sequence [24]. It is an extension of our computational combinatorial inhibitor design (CCLD) approach [29,30,31,32], which has been successfully used to design peptide inhibitors that could block the Ras interacting to its downstream target Raf protein [30,31,32].

In this work, we will first apply our approach to identify the epitopes of HA1 of H3N2 or H5N1 to three known antibodies such as AbHC19 [22], AbCR9114 [21] and AbBH151 [18], in comparison with the previous experimental results and crystal structures. Afterwards, we will predict the epitopes to the newly developed antibody 4F5. The predicted epitopes will be verified experimentally and used for the design of antibody therapeutics and vaccines of influenza viruses.

## 2. Materials and Methods

### 2.1. Homology Modeling of the Antibody 4F5

While the structures of antibodies HC19 [22], CR9114 [21] and BH151 [18] were taken from the crystal structures of their complex with antigen such as HA1 and HA2 of influenza virus, a homology model of antibody 4F5 was built as following. Firstly, the sequence of the antibody was used to search for the closest related antibody with known 3D structure using the BLAST (http://blast.ncbi.nlm.nih.gov) database search tool. The closest related proteins with known 3D structures suitable as modeling templates were found to be PDB [33] entry 2XZA (the structure of recombinant A.17 antibody Fab fragment [34]), entry 4EVN (the structure of Fab CR6261 [35]) and entry 4XVJ (the structure of antibody HC33.1 for HCV [36]) for VL domain, and entry 1W72 (the structure of antibody FAB-HYB3 [37]), entry 3EYQ (the structure of MJ5 Fab [38]), and entry 3QOS (the structure of human germline antibody 3-23/B3 [39]) for VH domain. While the VL domain of 4F5 show sequence identities of 91% to 2XZA, 94% to 4EVN, 92% to 4XVJ, the VH domain show sequence identities of 72% to 1W72, 68% to 3EYQ and 69% to 3QOS, respectively.

In a second step, a homology model of the antibody 4F5 was built using the templates described above. The model was created using the MODELLER software version 9.12 [40].

### 2.2. MCSS of Functional Groups

The MCSS method was originally developed to determine energetically favorable positions and orientations of functional groups in a target protein [25]. Using the homology model of an antibody, our quCBit software (http://www.medchemsoft.com), which implements our MCSS approach, was used to scan the preferred locations of functional chemical groups on the binding surfaces around the “complementarity determining regions” (CDRs). Eleven functional groups used corresponds to the side chains of different amino acids as listed in Table 1. The parameters for both protein and functional groups were taken from the CHARMM22 all-hydrogen atom force field [41].

Three hundred replicas of each functional group were randomly distributed inside a sphere with a 12 Å radius around residues of the CDRs of the antibodies. As the details of the CDR loop conformations have insignificant effect on the distribution of MCSS minima and on the sequence pattern derived from the minima, we use only single conformation of the CDR [24]. Table 2 lists the CDRs defined for the light and heavy chains of each antibodies. A 500-step MCSS was performed. During all the MCSS calculations, each replica only interacts with a target protein, but not with the other replicas. Details of calculations were described previously [24].

The nonbonded interaction was truncated at 20 Å. The dielectric constant was set to 10 to mimic solvent screening effects [42]. The choose of simple dielectric constant is based on the fact that the MCSS calculations presented here focus to obtain the optimal position of fragment minima on the surface of antibody, instead of accuracy of the protein-fragment binding energies. 

### 2.3. Identification of Sequence Pattern

By trial and error, interaction energy of −10.00 kcal/mol was used as the threshold for the minima of polar and apolar functional groups. For the positively charged groups MAMM and MGUA, as well as large group INDO, a threshold of −15.00 kcal/mol was used due to their strong interactions to the antibodies. These cutoffs of interaction energies give well-defined clustering of MCSS minima on the binding surface of antibody and clearly reveal the pattern of MCSS minima distribution.

The spatial patterns of the locations of the MCSS minima on the surface of the antibody were converted into a sequence pattern, served as the fingerprint to identify the epitopes of antigens.

### 2.4. Search for Epitopes Based on the Sequence Pattern

The sequence pattern obtained using the method described in 2.3 was used to identify the peptides derived from HA1. The details of the epitope searching method were described previously [24,43].

## 3. Results

### 3.1. Recognition of HA1 by Antibody HC19.

**Figure 1:**
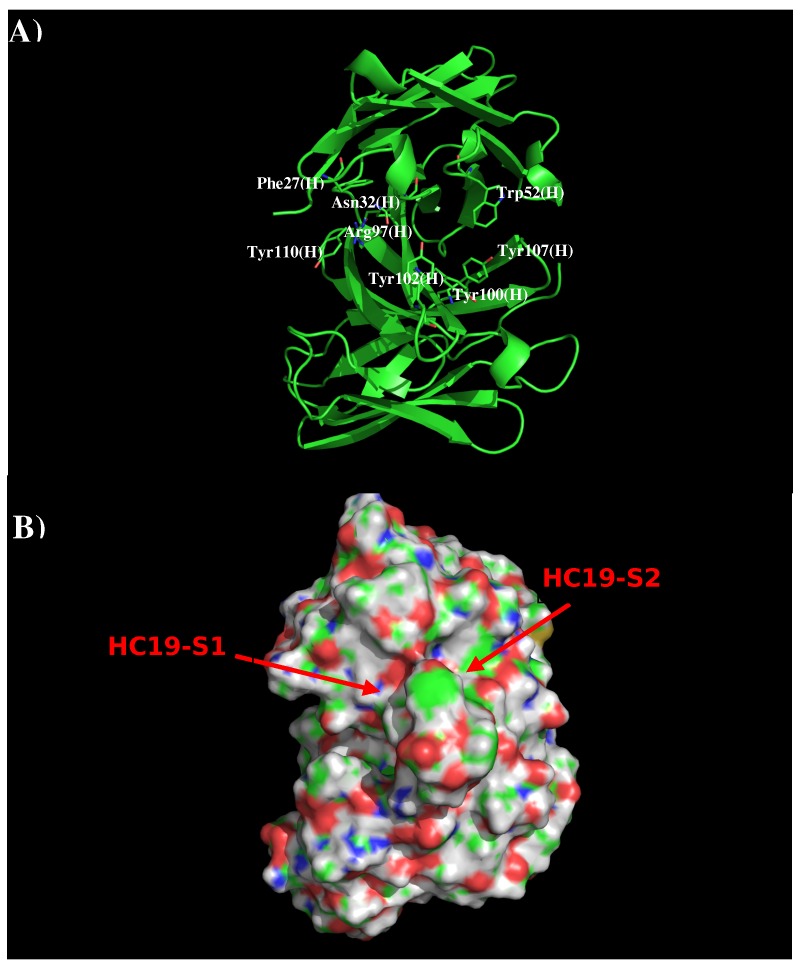
Model structure (**A**) and surface representation (**B**) of antibody HC19. Residues important for the antibody interactions are shown in stick form in (**A**). (L) and (H) denote the L and H chain of the antibody respectively. The figure was prepared using PyMOL [44].

Antibody HC19 has been developed against H3N2 influenza virus [22]. The crystal structure indicates that the antibody recognizes the receptor binding domain, thus blocking the entry to the host cell [19] (PDB code 2VIR). Figure 1 shows the structure and surface of AbHC19. There are two distinct regions around the CDR3 loop which are potentially significant to binding. The first region HC19-S1 is formed by residues Phe27, Asn32, Arg97, Tyr102, and Tyr110 of the H chain, with these residues contributing towards a polar charged surface. The second region HC19-S2 is formed by residues Trp52, Arg97, Tyr100, Tyr102, and Tyr107 also of the H chain. These residues form a surface with both polar and hydrophobic properties. These two binding surfaces (S1 and S2) are separated by ca. 9.0 Å.

**Figure 2:**
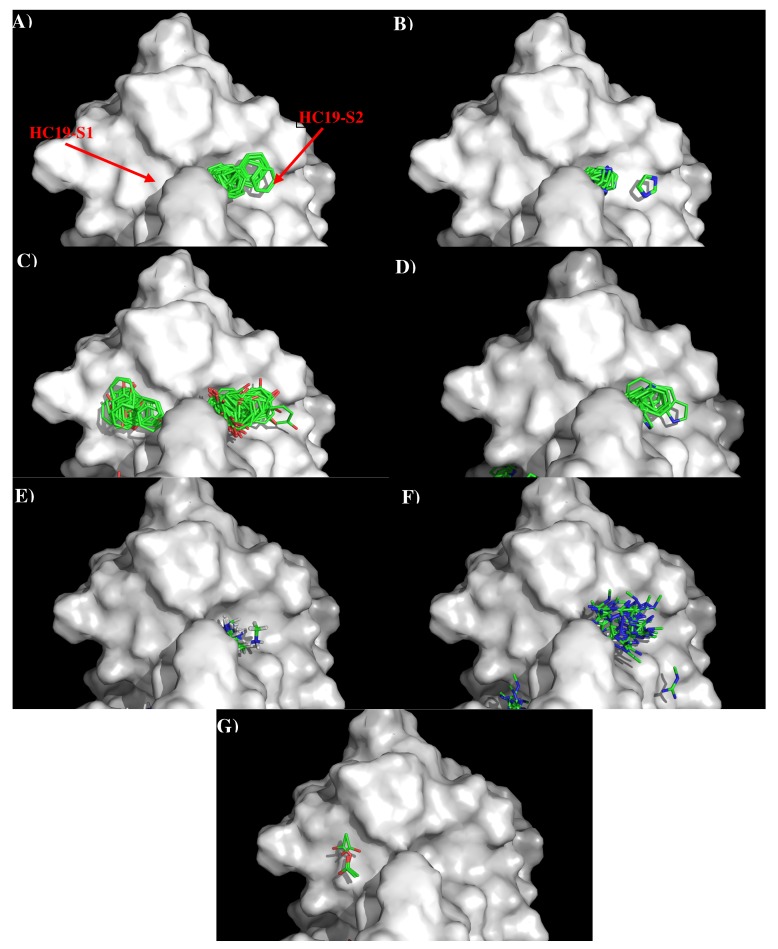
Selected MCSS minima of functional groups on the surface of antibody HC19. (**A**) BENZ; (**B**) IMIA; (**C**) PHEN; (**D**) INDO; (**E**) MAMM; (**F**) MGUA; (**G**) ACET. Figures were prepared using PyMOL [44].

Figure 2 shows the distribution of the MCSS minima of functional groups on the surface of AbHC19 occupying the two binding sites, S1 and S2. Because the binding pockets are quite deep, the minima are often buried deep inside the binding sites. Table S1 (Supplementary Data) shows the number of minima in each cluster, their interaction energies and any specific interactions with residues. Overall, a strong correlation was observed between the distribution of the MCSS minima and the physical properties of the antibody surface at S1 and S2. Generally, minima were only observed for functional groups that are charged or aromatic polar functional groups, apart from benzene. We can see from Table S1 (Supplementary Data) that most of the clusters occurred at the S2 binding site. Of the seven functional groups that formed minima, two formed clusters at S1, and six formed clusters at S2. Minima for both PHEN (16) and ACET (5) functional groups were observed, as we would expect forming polar interactions with the positively charged surface of S1, notably with Arg97(H) of the antibody. Generally, larger clusters consisting of minima of BENZ (112), IMIA (55), PHEN (7), INDO (33), MAMM (66), and MGUA (49) groups were observed at S2. Here we see a combination of both polar and hydrophobic interactions in keeping with the properties of the S2 surface. These interactions include hydrophobic interactions between groups (BENZ, IMIA, PHEN, INDO, MGUA) and Trp52(H), Arg97(H) of the antibody. Polar interactions were also observed between groups (INDO, MAMM, MGUA) and Asp98(H), Arg 97(H) of the antibody. The interaction energies of the minima are shown in Table S1 (Supplementary Data). These values are generally as expected, with the polar and non-polar groups showing interaction energies of around −10.00 kcal/mol, and the charged and IMIA groups of around −15.00 kcal/mol, in line with the defined cut-offs. There are some exceptions, PHEN and MGUA groups both show stronger interaction energies, with Arg97(H) (−17.60 kcal/mol) and Tyr107(H) (−18.70 kcal/mol), respectively. The higher concentration of interactions that we observe between the functional groups and the S2 surface are probably because S2 has the properties of being both charged and hydrophobic. This allows the site to interact with a greater variety of functional groups.

Using the minima on the two surfaces S1 and S2, we constructed a sequence pattern for the peptides that could potentially bind to the antibody. The maximum distance between the two binding surfaces S1 and S2 is approximately 9.0 Å, corresponding to a separation by one amino acid between potential residues of antigen bind to residue at S1 and S2 of antibody. Our results showed most of the functional group minima were located at S2, with only PHEN and ACET groups found at surface S1. Therefore, the key sequence pattern for the binding epitope peptides can be defined as “X-J”, in which X = R, K, Y, W, H, F, “-” = any amino acid and J = D/E, Y. Note that the sequence is aligned from S2 to S1 as the negatively charged minima ACET is only found on S1, thus corresponding to the C-terminal of peptides. Table 3 lists the distribution of key MCSS minima and the derived amino acid sequence pattern.

**Figure 3:**
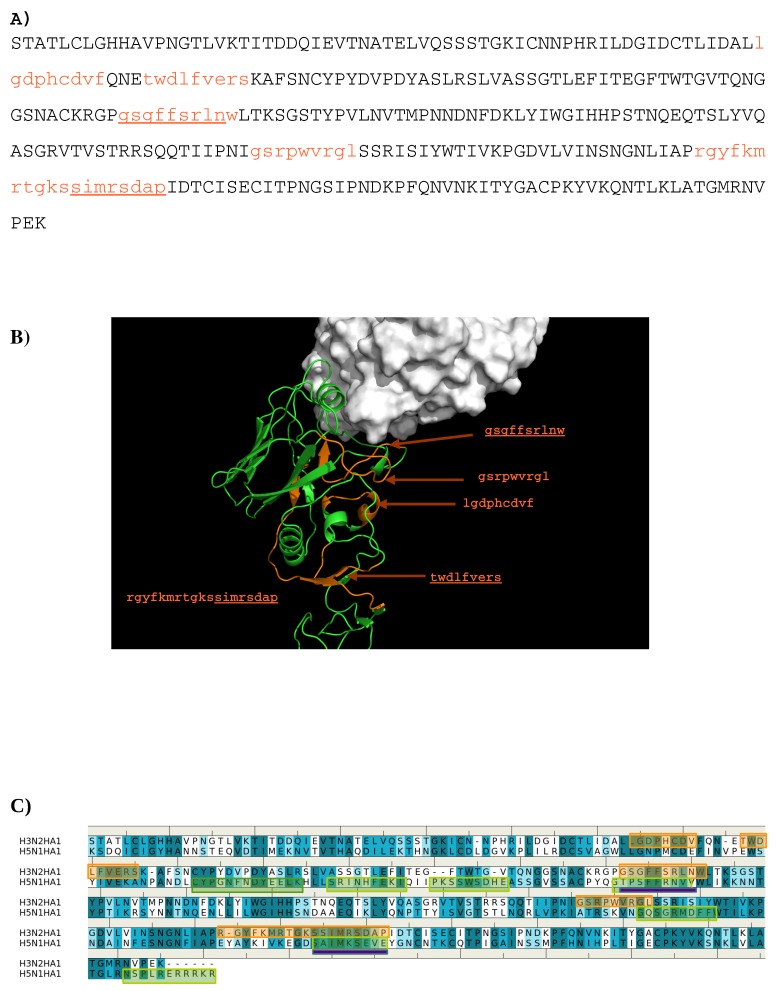
(**A**) the predicted epitopes of H3N2 HA1 that binds to antibody HC19. The epitopes are highlighted in lower case and colored orange in the protein sequence. (**B**) Backbone representation of the antigen H3N2 HA1 showing the predicted epitopes in orange and the antibody in surface. (**C**) Sequence alignment of HA1 protein of influenza viruses H3N2 and H5N1. The boxes highlight the epitopes predicted for H3N2 (**orange**) and H5N1 (**green**). The overlay regions between two subtypes are underlined in purple. Figures were prepared using PyMOL [44].

The sequence pattern was subsequently used to search for “binders” from the peptide libraries derived from the sequence of HA1 from the H3N2 virus (see Methods section). The seven libraries were searched for peptides matching the calculated sequence pattern of the binding epitope. Five peptides of HA1 were predicted to bind to the antibody (**1-** “lgdphcdvf”; **2-** “twdlfvers”; **3-** “gsgffsrlnw”; **4-** “gsrpwvrgl”; **5-** “rgyfkmrtgkssimrsdap”). Figure 3 shows the amino acid sequence of HA1 with the predicted epitopes highlighted in lower case and colored in orange (Figure 3A), and their location in the protein structure (Figure 3B). For comparison, the predicted epitopes of HA1 from the H5N1 virus are also shown (Figure 3C). Figure 3B reveals that peptide **3** is located in the interaction site, and is clearly involved in the HA1-antibody binding. Close inspection show residues Ser145 and Trp153 of peptide **3** interacting with Asp101 and Tyr102 of the H chain, respectively. These direct contacts between the antigen and antibody are located in the regions of residues 134–144, residues 162–168 and residues 173–203 of HA1 of H3N2. The Tyr102, of the above-mentioned Trp153-Tyr102(H) interaction, is one of the residues involved in specific interactions identified from our MCSS calculations. This interaction occurs at the center of the interface, potentially playing a significant role in the antibody-antigen recognition. Peptide **1** is also of interest as a potential epitope. This peptide is located at the vestigial esterase domain, another known antibody recognition region.

Epitope mapping was also performed for HA1 from the H5N1 virus. Figure 3C shows the comparison of the predicted epitopes between two viruses. Only two regions are identified to be overlay between the two antigens (“gssffsrlnw” vs. “gtpsffrnvv”, “sstmrsdap” vs. “satmkseve”). These are underlined in Figure 3A. The epitopes from H5N1 do not include the important Trp153-Tyr102(H) interaction observed between H3N2 and the antibody as Trp153 of H5N1 is outside of the identified epitope “gtpsffrnvv”. This is in keeping with the selectivity shown experimentally of AbHC19 towards H3N2 [34,35,44].

### 3.2. Recognition of HA1 by Antibodies CR9114 and FI6V3.

**Figure 4:**
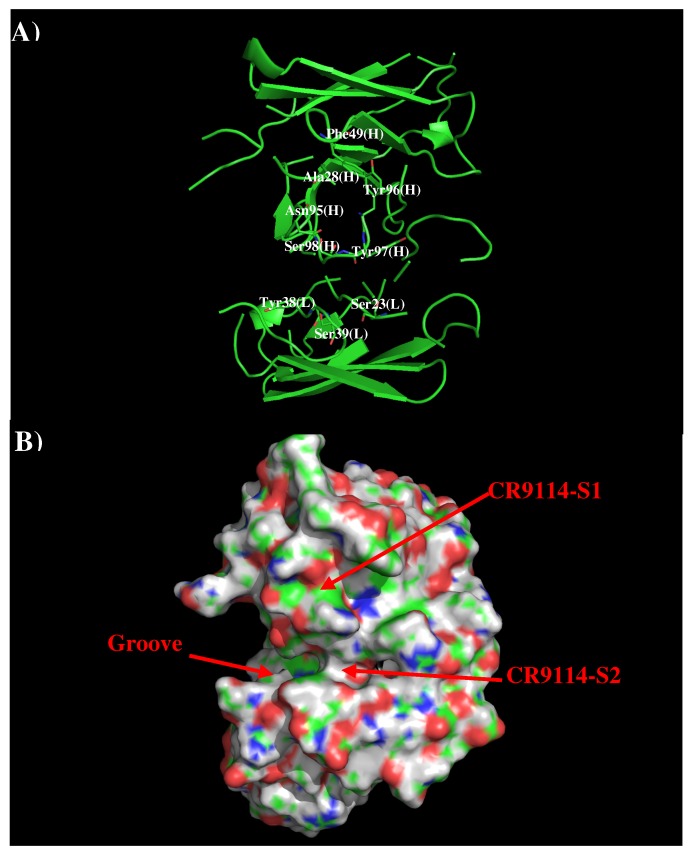
Model structure (**A**) and surface representation (**B**) of antibody CR9114. The important residues for the antibody interaction are shown in stick form in (**A**). (L) and (H) denote the VL and VH domain of the antibody respectively. The figure was prepared using PyMOL [44].

Antibodies CR9114 and FI6V3 have been developed against H3N2 influenza virus [21,36]. The crystal structures of their complexes to H3N2 indicated that both antibodies bind in similar fashion to the membrane fusion part of HA. Therefore, we only present the results of AbCR9114 here. Figure 4 shows that structure and surface of AbCR9114. There are two distinctive regions around the CDR3 loop. The first site (CR9114-S1) is a hydrophobic surface formed by residues Ala28, Phe49, Asn95, Tyr96, and Tyr97 of H chain while the second polar surface (CR9114-S2) is formed by residues Ser23, Tyr38, and Ser39 of L chain and Ser98 of H chain. These two binding surfaces are separated by ca. 11.50 Å.

**Figure 5:**
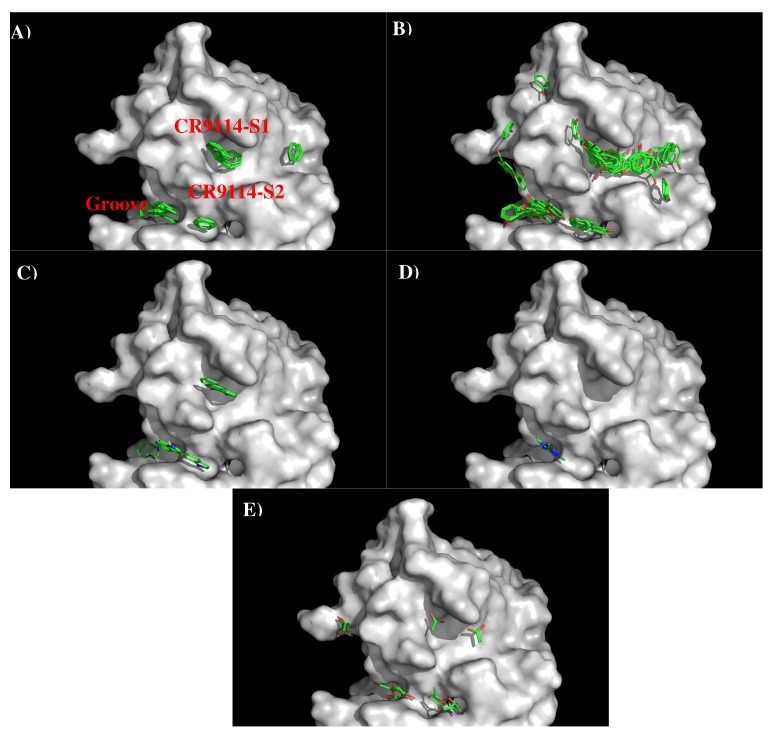
Selected MCSS minima of functional groups on the surface of antibody CR9114. (**A**) BENZ; (**B**) PHEN; (**C**) INDO; (**D**) MGUA; (**E**) ACET. Figures were prepared using PyMOL [44].

Figure 5 shows the locations of the minima of functional groups on the surface of AbCR9114. Table S2 (Supplementary Data) shows the number of minima in each cluster and their interaction energies with residues. The minima are mainly clustered around the S1 and S2, as well as a groove nearby. No minima of small groups such as MEOH, MESH, and IBUT were found on the surfaces around the CDR3 loop. For the apolar groups, 29 and 21 BENZ minima were found at the S1 and S2 with favorable interaction energy values of −11.10 kcal/mol and −10.40 kcal/mol, respectively. The stronger interaction at S1 is due to the hydrophobicity of the binding site formed by two Tyrosine residues (Tyr96 and Tyr97 of H chain). For PHEN group, the minima is more spread out. 42 PHEN minima were located at S1 and nearby the surface, half of which concentrated at S1 with strong interaction energies up to −13.30 kcal/mol. These minima form interaction to Phe49 of H chain. Only seven minima were found at S2 with the binding energy range of (−10.00, −11.30) kcal/mol. For the INDO group, four minima were found to overlay each other at S1 with binding energies of ca. −15.40 kcal/mol. These minima form interaction to Phe49(H) and are oriented perpendicularly to Tyr96(H). Three INDO minima were found at S2, one of which formed a hydrogen bond to Asn25(L) with energy of −15.50 kcal/mol and the other two minima forming similar interaction to Tyr38 of L chain (−15.20 kcal/mol). For the charged groups, no MAMM minima were found around CDR3 loop while four MGUA minima were found only at S2 with energy range of (−15.00, −17.20) kcal/mol arising from its electrostatic interaction to Asp101(H). For the ACET group, four minima were found at S1, one of which interacts with Ser46(H) and three interacting with Ser51(H) showing energy range of −17.20 kcal/mol and (−15.20, −15.40) kcal/mol, respectively. At S2 six ACET minima were found, two of which interact in a similar fashion with Ser39(L) with energy of −16.10 kcal/mol, while other four minima interact with Arg22(L) showing energy range of (−15.10, −18.10) kcal/mol.

**Figure 6:**
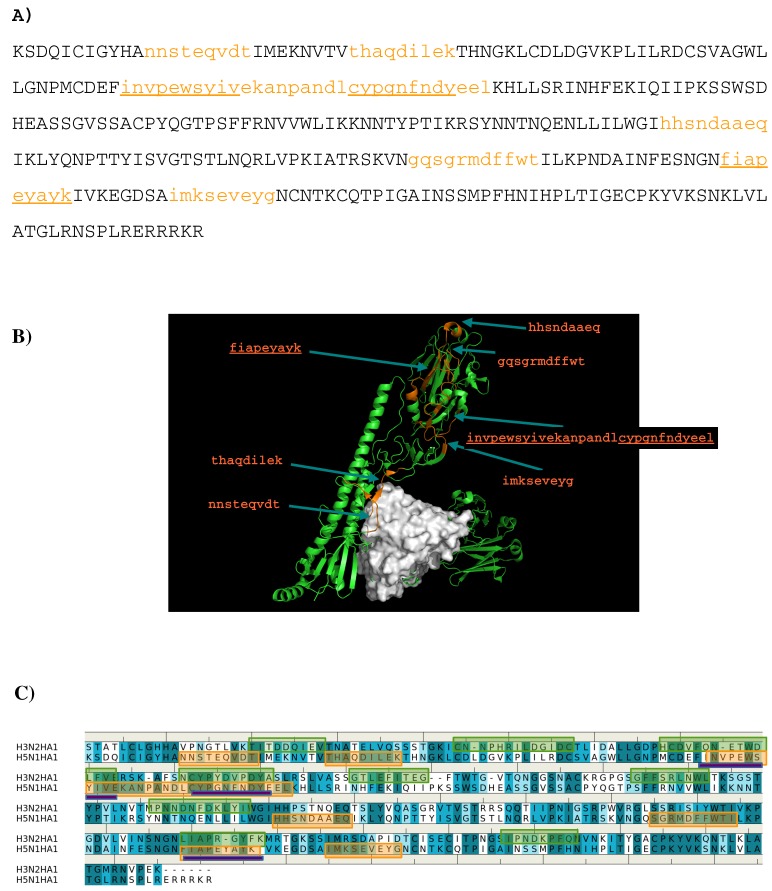
(**A**) the predicted epitopes of H5N1 HA1 (**A**) binding to antibody CR9114. The epitopes are highlighted in lower case and colored orange in the protein sequence. (**B**) Backbone representation of the antigen H3N2 HA1 showing the predicted epitopes in orange and the antibody in surface. (**C**) Sequence alignment of HA1 protein of influenza viruses H3N2 and H5N1. The boxes highlight the epitopes predicted for H3N2 (**green**) and H5N1 (**orange**). The overlay regions between two subtypes are underlined in purple. Figures were prepared using PyMOL [44].

Based on the distribution of the important minima as shown in Figure 6, a sequence pattern for peptides that bind to AbCR9114 was derived. The MCSS minima at the binding sites S1 and S2 are separated by ca 11.50 Å, a distance that could accommodate two amino acids. While most of the apolar and negatively charged group (i.e., BENZ, PHEN, INDO and AET) are located at both surfaces, only positively charged group MGUA was identified at S2 by interacting to residue Asp101(H). Therefore, the key sequence pattern for the binders was derived as “X-J”, in which X = R,F,W,Y,D/E, and J = F,W,Y,D/E. Table 4 lists the distribution of key MCSS minima and the derived sequence pattern.

Seven peptides (I- “nnsteqvdt”, II- “thaqdilek”, III-”invpewsyivekanpandlcypgnfndyeel”, IV- ”hhsndaaeq”, V- “gqsgrmdffwt”, VI- ”fiapeyayk”, VII- “imkseveyg”) are predicted to bind to the antibody (Figure 6A) and Figure 6B) shows the location of these peptide in the crystal structure of AbCR9114 complexed with HA of H5N1. Peptide VI is buried inside the protein so as to be discarded as epitope. Of all the peptides, peptide II is located at the interface between the antibody and HA1 of H5N1 virus, together with peptide I forms a recognition surface. Two more regions were also predicted and are formed by the C-terminal portion of peptide III and peptide VII, and by peptide IV and N-terminal portion of peptide V, respectively. These two regions are located close to the vestigial esterate domain and the receptor binding domain, respectively. Figure 6C shows the comparison between the epitopes predicted for the two viruses binding AbCR9114. Only the region of peptide III is consistently observed in both antigens as the other overlay region at the peptide VI is buried in the protein. The epitope “hcdvfqnetwdlfv” of subtype H3 is located at the vestigial esterate domain.

### 3.3. Recognition of HA1 to Antibody BH151

**Figure 7:**
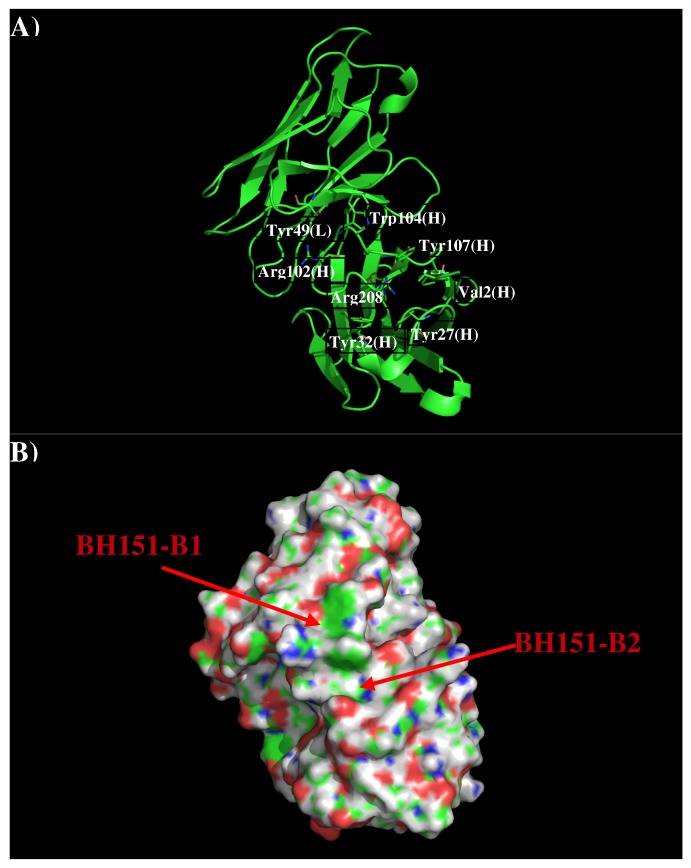
Model structure (**A**) and surface representation (**B**) of antibody BH151. Residues for the antibody interactions are shown in stick representation in (**A**). (L) and (H) denote the VL and VH domain of the antibody respectively. The figure was prepared using PyMOL [24].

AbBH151 is developed to against H3N2 [18]. The crystal structure revealed that AbBH151 binds to a vestigial esterase domain. Figure 7 shows that structure and surface of AbBH151. The surface around the CDR3 loop is highly charged with a binding site (BH151-S1) formed by residues Val2, Tyr27, Tyr32, Arg98, Arg102, Phe105 and Tyr107 of H chain, and a relatively flat surface (BH151-S2) formed by residues Tyr49 of L chain and Arg102, Tyr104 of H chain. The two surfaces are separated by 9.50 Å.

**Figure 8:**
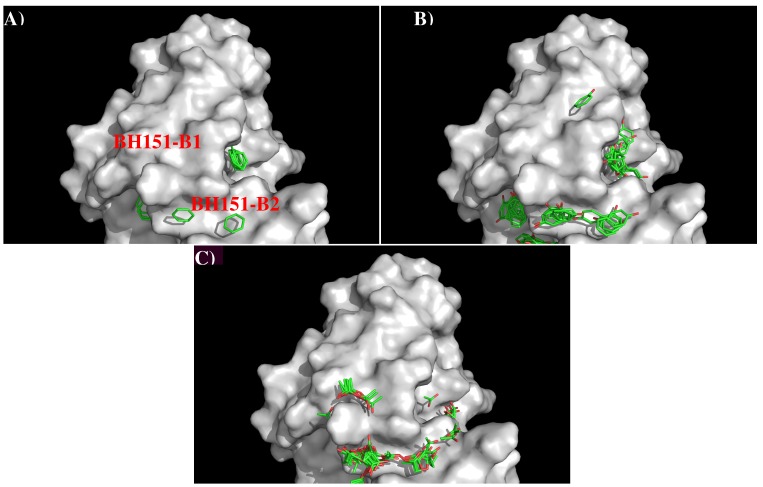
Selected MCSS minima of functional groups on the surface of antibody BH151. (**A**) BENZ; (**B**) PHEN; (**C**) ACET. Figures were prepared using PyMOL [24].

Figure 8 shows the locations of the minima for the functional groups on the surface of AbBH151. Table S3 (Supplementary Data) shows the number of minima in each cluster and their interaction energies with residues. A relatively small number of minima are clustered around CDR3 loop, including BENZ, PHEN, and ACET. While two BENZ minima, 11 PHEN minima and 30 ACET minima were found at the binding site B2, only 9 ACET minima were located at the B1. For the BENZ group, two minima are separated by 8.00 Å along the site B2 and interact with Phe105(H) with energies of −10.10 kcal/mol and −10.20 kcal/mol, respectively. The same positions were occupied by five and six PHEN minima with interaction energy range of (−10.00, −10.40) kcal/mol and (−10.00, −11.70) kcal/mol, respectively. The second group forms stronger interactions with Arg98(H) at the bottom of the binding site. For the ACET group, 30 minima at B2 are split into two clusters, one interacts with Arg102(H) measuring energies up to −17.70 kcal/mol, and the other one interacts with Arg98(H) showing energies up to −18.50 kcal/mol. At the binding site B1, nine ACET minima mainly interact with Arg102(H) (energy up to −16.80 kcal/mol).

Based on the distribution of the important minima as shown in Figure 8, a sequence pattern for peptides that bind to AbBH151 was derived. The MCSS minima at the binding sites B1 and B2 are separated by ca 9.50 Å, implying one amino acid insertion. As only ACET minima were obtained at B1 while ACET, BENZ and PHEN minima were found at B2, the key sequence pattern for the binders was defined as “X-J”, in which X = D/E, and J = F,Y,D/E. The sequence is aligned from B1 to B2 as more ACET minima were located at B2, corresponding to the C-terminal of peptides. Table 5 lists the distribution of key MCSS minima and the derived sequence pattern.

**Figure 9:**
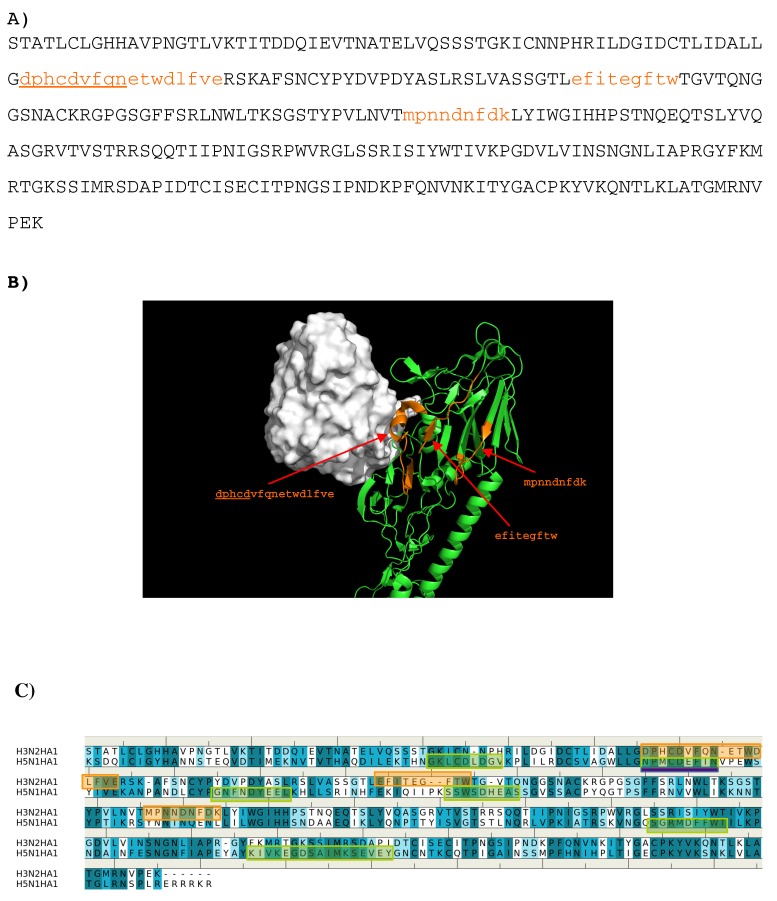
(**A**) the predicted epitopes of H3N2 HA1 (**A**) binding to antibody BH151. The epitopes are highlighted in lower case and colored orange in the protein sequence. (**B**) Backbone representation of the antigen H3N2 HA1 showing the predicted epitopes in orange and the antibody in surface. (**C**) Sequence alignment of HA1 protein of influenza viruses H3N2 and H5N1. The boxes highlight the epitopes predicted for H3N2 (**orange**) and H5N1 (**green**). The overlay regions between two subtypes are underlined in purple. Figures were prepared using PyMOL [44].

Overall, only three peptides (A: “dphcdvfqnetwdlfve”, B: ”efitegftw”, C: ”mpnndnfdk”) from H3N2 HA1 are predicted to bind to the antibody. Figure 9 shows the location of these epitopes. Out of these, the first half (“dphcdv”) of the peptide A forms a helical conformation and interact to the CDR3 loop of the antibody (residues 208–217 of H chain). This epitope is also obtained for the H5N1 HA1 as shown in Figure 9C.

### 3.4. Prediction of Epitopes of HA1 to Antibody 4F5

**Figure 10:**
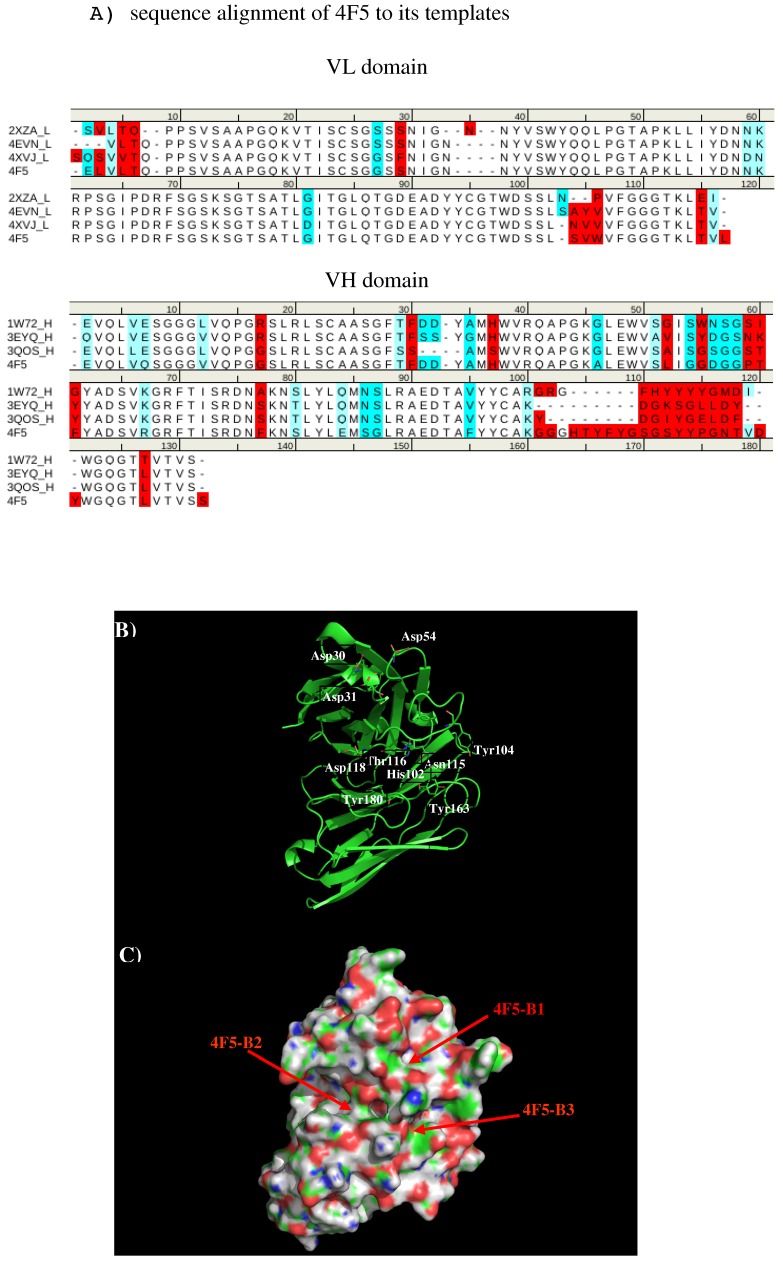
(**A**) Sequence alignment of VL domain of 4F5 to its templates 2XZA [34], 4EVN [35], and 4XVJ [36] for VL domain, and VH domain of 4F5 to its templates 1W72 [37], 3EYQ [38], and 3QOS [39]. (**B**) The model structure of antibody 4F5. The important residues for the antibody interaction are shown in stick representation in (**A**). (L) and (H) denote the VL and VH domain of the antibody respectively. (C) surface representation of antibody 4F5. The figure was prepared using PyMOL [44].

We created a 3D model of the VL and VH domains of antibody 4F5 [23] using the X-ray structures with PDB [33] identifiers 2XZA [34], 4EVN [44], and 4XVJ [36] as templates for VL domain, and identifiers 1W72 [37], 3EYQ [38] and 3QOS [39] for VH domain. Figure 10 shows the sequence alignment of 4F5 to its templates, model structure and surface representation of Ab4F5 with the important residues highlighted. Compared to other antibodies, the surface around the CDR3 loop are highly charged with one site (4F5-B1) close to a cluster of negatively charged residues Asp31, Asp40, and Asp54 of L chain, and another site (4F5-B2) formed by residues His102 of L chain, residues Thr30, Thr50, and Asp51 of H chain, as well as a more hydrophobic site formed by residues His102, Tyr104, and Asn115 of L chain and Tyr33 of H chain. These three sites are separated by ca. 13.00 Å from each other.

**Figure 11:**
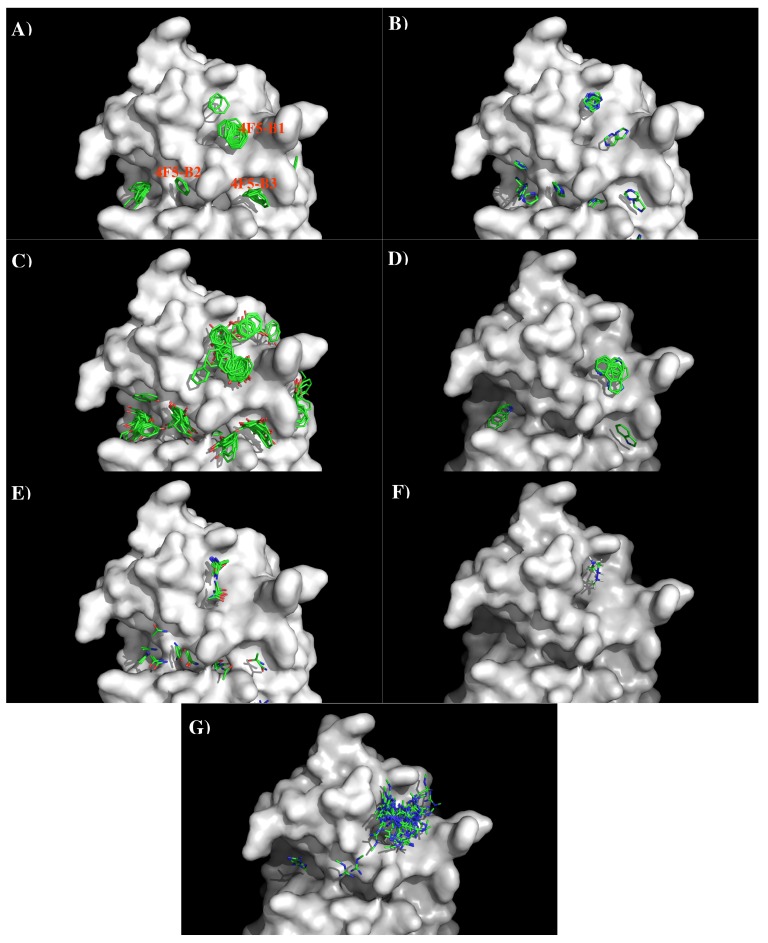
Selected MCSS minima of functional groups on the surface of antibody 4F5. (**A**) BENZ; (**B**) IMIAl (**C**) PHEN; (**D**) INDO; (**E**) ACEM; (**F**) MAMM; (**G**) MGUA. Figures were prepared using PyMOL [44].

Figure 11 shows the locations of the minima of functional groups on the surface of antibody 4F5. Overall, the minima are clustered around the three binding sites B1, B2, and B3 as defined above. Table S4 (Supplementary Data) shows the number of minima in each cluster and their interaction energies with residues. For the aromatic group, clusters of 37, 6, and 11 BENZ minima were found at the three sites with favorable energies of −11.00 kcal/mol (B1), −10.00 kcal/mol (B2) and −11.20 kcal/mol (B3), respectively. Minima at these sites form hydrophobic interactions with Leu47(H), to Tyr50(H) and with both Tyr33(H) and Tyr104(L). For the IMIA group, 10, 3, and 3 minima were found at three sites with favorable energies of −12.90 kcal/mol (B1), −10.70 kcal/mol (B2), and −10.90 kcal/mol (B3), respectively. These minima interact with Leu47(H), Asp108(L), and with both Tyr33(H) and Tyr104(L), respectively. For the PHEN group, 37, 20, and 18 minima were found with energies up to −13.40 kcal/mol (B1), −13.80 kcal/mol (B2) and −13.40 kcal/mol (B3). While the best minimum at the site B3 forms hydrogen bonds with Asn115(L) and Asn32(H), the best minimum at B2 forms pi-pi interaction with both Tyr104(L) and Tyr33(H). For the INDO group, 11 minima were found at the B1 with favorable energies of −16.10 kcal/mol by hydrogen bonding to CO group of Gly101(L). Only one minimum was found at B3 with energy of −15.20 kcal/mol. For the case of polar groups, 16, 4, and 2 ACEM minima were found at three sites with favorable energies of −11.70 kcal/mol, −10.00 kcal/mol, and −10.10 kcal/mol, respectively. At these three sites, the best minimum interacts with Asp54(L), Asp51(H), and Asn32(H). The positively charged minima MAMM and MGUA were found only at B1 by interacting with Asp54 of L chain. While three MAMM minima have the interaction energies of (−15.20, −16.00) kcal/mol, the best minima consisting of 60 MGUA fragments has the strongest interaction energy of −20.80 kcal/mol.

Based on the distribution of the important minima as shown in Figure 11, a sequence pattern for peptides that bind to Ab4F5 was derived. As the binding B2 is ca. 15.00 Å distant from the CDR3 loop, we disregard the minima at this binding site. The MCSS minima at the binding sites B1 and B2 are separated by ca 13.00 Å, a distance that could accommodate two amino acids. Based on the distribution of MCSS minima, the key sequence pattern for the binders was defined as “X-J”, in which X = R/K,Q/N,W,F/Y/H, J = F/Y/H,W. Table 6 lists the distribution of key MCSS minima and the derived sequence pattern.

**Figure 12:**
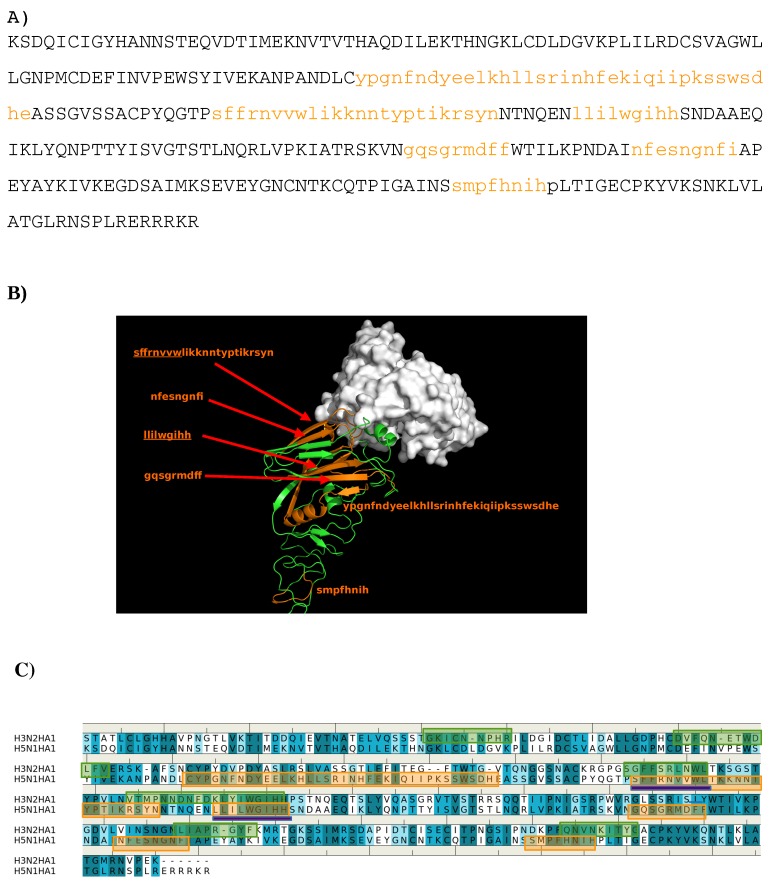
(**A**) the predicted epitopes of H5N1 HA1 (**A**) to antibody 4F5. The epitopes are highlighted in lower case and colored orange in the protein sequence. (**B**) Backbone representation of the antigen H5N1 HA1 showing the predicted epitopes in orange and the antibody in surface. (**C**) Sequence alignment of HA1 protein of influenza viruses H3N2 and H5N1. The boxes highlight the epitopes predicted for H3N2 (green) and H5N1 (orange). The overlay regions between two subtypes are underlined in purple. Figures were prepared using PyMOL [44].

The predicted epitopes of the H5N1 HA1 using the above-mentioned protocol are shown in Figure 12A) with the epitope shown in orange lower case characters. Six binding peptides (E1: “ypgnfndyeelkhllsrinhfekiqiipksswsdhe”, E2: ”sffrnvvwlikknntyptikrsyn”, E3: ”llilwgihh”, E4: ”gqsgrmdff”, E5: ”nfesngnfi”, E6: ”smpfhnih”) are predicted from H5N1 HA1 to bind to the antibody (Figure 12B). While the peptide E1 and E6 are binding to the HA2 in a similar fashion as observed in the crystal structure of AbCR9114 complexed with H5N1 (Figure 12C), peptides E3 and E4 are buried inside the protein. Therefore, the epitopes for the antibody recognition are considered to be peptides E2 and E5 which are locate at the receptor binding domain, similar to those that bind to antibody HC19 [18,22]. This is in contrast to recent phase display experiments in which a conserved epitope (WLLGNP) amongst different clades was identified; this peptide is located at the vestigial esterase domain where the antibody BH151 binds [23]. Figure 12C) also shows the epitopes predicted from H3N2 HA1 and its comparison to H5N1 HA1. While the overlay region **E3** (“llilwgihh” vs. “lyiwgihh”) is buried in protein, the other region at **E2** (“sffrnvvw” vs. “gffsrlnwl”) is close to the second binder peptide “dvfqnetwdlfv” which is located at the vestigial esterase region and next to the position of 76-WLLGNP-81 identified as epitope in H5N1. Therefore, Ab4F5 could recognize the H3N2 by binding to the vestigial esterase domain.

## 4. Discussion

Previously, a new method has been developed to predict the epitopes of antigens that bind to specific antibodies, via three steps: 1) mapping of functional groups onto the surface of the antibody, 2) deriving a sequence pattern for potential binding peptides based on the distribution of significant minima of functional groups, and 3) searching the antigen sequence for binding peptides which are consistent with the sequence pattern [25,26,27,31]. Here we applied this approach to identify the recognition of antibodies to influenza virus, and predict the epitopes for the newly developed 4F5 antibody to H5N1 virus [23]. This could be useful for the development of new antibody therapies and vaccines.

The crystal structures of currently available antibodies targeting the HA of influenza viruses revealed that three regions of epitopes for antibody binding are known: (I) proximate to membrane fusion (e.g., AbCR6116 [22], AbCR9114 [21]), (ii) at the receptor binding domain (e.g., AbHC19 [22]) and (iii) at the vestigial esterase domain (e.g., AbBH151 and AbHC45 [18]). Our calculations have qualitatively reproduced the recognition regions of these antibody–antigen complexes and provided additional insights for the origins of the binding specificity binding. For AbHC19, five peptides from HA1 of H3N2 are predicted to be the binders with the peptide 3 (residue 144–152) is in fact part of the observed epitopes (Figure 3C). A direct binding between residue Trp153 of the HA1 and Tyr102 of heavy chain of antibody is located at the center of antibody-antigen interface. This interaction could play a controlling role in the recognition of AbHC19 to H3 subtypes of influenza virus as it is not found from H5N1 HA1 (Figure 3C). In addition, peptide 1 (residue 71–79) is predicted at the vestigial esterase domain (Figure 3A). This epitope is also missed from the epitope mapping of H5N1 (Figure 3B). Therefore, AbHC19 is considered to be selective to the H3 subtype with respect to H5, in agreement to the experimental observation [34,35,44].

For the antibody CR9114, our predicted epitopes indicated three important features. Firstly, epitopes I (residues 26–34) and II (residues 43–51) from H5N1 HA1 are close to the membrane fusion domain. These epitopes count for two-third of recognition regions revealed from the crystal structure of the complex (Figure 6). Secondly, epitopes III (residues 87–117) and VII (residue 280–288) form a recognition surface at the vestigial esterase domain, while the epitopes IV (residue 195–203) and V (residue 237–247) are at located the receptor binding region. These two regions are recognized by AbCR8071 and AbCR8033 [20], respectively. Thirdly, the epitopes predicted from H3N2 HA1 are located at the receptor binding domain only, so that the observed cross reactivities of AbCR9114 to subtypes H1, H3, H7, and H9 [21] could adopt different binding mechanism, such as that similar to AbHC19 or AbBH151, depending on the antigen subtype.

As shown in Figure 9, the recognition of antibody BH151 to H3N2 is better defined as only three epitopes were obtained with one at the recognition regions identified from the crystal structure. However, the other two are not close to the interface (Figure 9B). The epitope at the interface of crystal structure is also identified as the binding peptide in H5N1, indicating that the AbBH151 could bind to H5N1. As AbBH151 is considered to specifically neutralize HA from the H3N2 [34], it is possible that the binding to the vestigial esterase domain in H5N1 is relatively weaker than the binding in H3N2. Further experimentation is required to verify our prediction.

As for the newly developed antibody 4F5 for H5N1, the predicted epitopes indicate its binding to H5N1 is at the receptor binding domain (Figure 12). Our results are consistent to the recent description of antigenic architecture of the hemagglutinin of H5N1 in which the major epitope regions for neutralizing antibody are concentrated at the receptor binding domain and membrane fusion [37]. However, this is in contrast to the previous study in which a conserved peptide 76(WLLGNP)81 amongst different H5 clades was proposed as binding pocket for the antibody 4F5 [23]. This sequence forms a helix at the vestigial esterase domain where the antibody BH151 binds to H3N2 (Figure 9C) [18]. Recent epitope mapping studies of an humanized antibody 8A8 on H5N1 has demonstrated that virus neutralization mediated by receptor binding domain blocking relies on the conformational epitope while binding to the linear epitope contributes to the neutralization by inhibiting membrane fusion [45]. Therefore, the antibody 8A8 neutralizes H5N1 through two mechanisms [45]. As our calculations are based on three dimensional structures of the antibody, the epitopes predicted here could be conformational character while the conserved peptide derived from phase display could be the linear type. The antibody 4F5 thus could bind two types of epitopes of H5N1 HA1, leading to virus neutralization via two mechanisms. Nevertheless, a recognition region (seq. “dvfqnetwdlfv”) at the vestigial esterase domain of H3N2 is predicted to be epitope of H3 subtype of influenza virus to 4F5, while the receptor binding domain is not. Further experiments are highly desirable to clarify these findings.

## 5. Conclusions

In the study presented here, we have applied a new developed method [31] to identify the antibody recognition regions of HA1 to several known antibodies and the newly developed antibody 4F5. Overall, our approach provides a qualitative assessment of peptide sequences from an antigen that could bind to antibody which is useful to reduce the amount of experimental work necessary to identify an antibody-binding epitope. Further development will rely upon the docking of antibodies to the antigen guided by the predicted epitopes.

While our calculations have reproduced the epitopes where antibodies HC19, CR9114 and BH151 bind, additional epitopes have been suggested for experimental verification. As demonstrated recently, a mAb binds to two types of epitopes of H5N1 HA1 and neutralize the virus via two mechanisms [45]. For the antibody 4F5, the epitopes of H5N1 and H3N2 are predicted at the receptor binding domain and at the vestigial esterase domain, respectively. Taking into account the recent results in which a conserved peptide 76(WLLGNP)81 is identified as the epitope of H5N1 [32], the neutralization of antibody 4F5 to H5N1 could thus also bind via different binding mechanisms to different epitopes.

## Figures and Tables

**Table 1:** Relationship between the functional groups used and amino acids.

	Functional group	Abbreviation	Amino Acids
Charged (-)	Acetate ion	ACET	ASP, GLU
Charged (+)	Methylguanidinium	MGUA	ARG
Charged (+)	Methylammonium	MAMM	LYS
Polar	Acetamide	ACEM	ASN,GLN
Polar	Methanol	MEOH	SER,THR
Hydrophobic	Methanethiol	MESH	CYS,MET
Aromatic Polar	Phenol	PHEN	TYR
Aromatic Polar	Indole	INDO	TRP
Aromatic Polar	Imidazole	IMIA	HIS
Aromatic Hydrophobic	Benzene	BENZ	PHE
Hydrophobic	Ibutane	IBUT	VAL, ILE, LEU, ALA

**Table 2:** Definition of binding sites for each antibody, based on our MFMD calculations.

Antibodies	L Chain	H Chain
AbHC19	Asn33, Tyr34, Asn36, Asn55,Pro58, Trp93	Asn32, Trp52,Ala53, Arg97, Asp98, Trp100, Tyr107
AbCR9114	Arg31,Tyr36,Tyr49,Pro55,Trp91	Asn25,Phe48,Ser50,Arg91, Asn94,Tyr96,Ser97
AbBH151	Ser30,Tyr49,Ala55,Arg91,Ser92,Tyr94	Tyr32,Phe33,Asn57,Arg98, Gly101,Arg102
4F5	Tyr32,Phe59,Gly101,His102,Tyr104,Phe105,Tyr106,ser108,Tyr112	Tyr33,Asp51,Pro56

**Table 3:** Distribution of key minima and the derived sequence pattern for the binding epitope peptides to antibody HC19. A sequence pattern of “X-J” [X = R/K,F,W,Y and J = (Y,D/E) ] was obtained.

Binding Surface	S2	9.00 Å	S1
**MCSS Minima Pattern**	BENZ		PHEN
	PHEN		ACET
	INDO		
	MAMM		
	MGUA		
**Sequence Pattern**	F	Gap of one amino acid	Y
	Y		D/E
	W		
	W		
	R/K		

**Table 4:** Distribution of key minima and the derived sequence pattern for the binding epitope peptides to the antibody CR9114. A sequence pattern of “X-J” (X = R,FW,Y,D/E, and J = F, W,Y,D/E) was obtained.

Binding Surface	B1	11.50 Å	B2
**MCSS Minima Pattern**	BENZ		BENZ
	PHEN		PHEN
	INDO		INDO
	MGUA		ACET
	ACET		
**Sequence Pattern**	F	Gap of 2 amino acid	F
	Y		Y
	W		W
	R		D/E
	D/E		

**Table 5:** Distribution of key minima and the derived sequence pattern for the binding epitope peptides to the antibody BH151. A sequence pattern of “X-J”(X = D/E, and J = F, Y,D/E) was obtained.

Binding Surface	B1	9.50 Å	B2
**MCSS Minima Pattern**	ACET		BENZ
			PHEN
			ACET
	
	
**Sequence Pattern**	D/E	Gap of one amino acid	F
			Y
			D/E
	
	

**Table 6:** Distribution of key minima and the derived sequence pattern for the binding epitope peptides to the antibody 4F5. A sequence pattern of “X-J”(X = R/K,Q/N,W,F/Y/H, and J = F/Y/H, W) was obtained.

Binding Surface	B1	13.00 Å	B2
**MCSS Minima Pattern**	BENZ		BENZ
	IMIA		IMIA
	PHEN		PHEN
	INDO		INDO
	ACEM		
	MAMM		
	MGUA		
**Sequence Pattern**	F	Gap of two amino acid	F
	H		H
	Y		Y
	W		W
	Q/N		
	R/K		

## Supplementary Materials

The following are available online at http://www.mdpi.com/2073-4468/8/1/2/s1, Table S1: Distribution of MCSS minima of functional groups on the binding surface of AbHC19, Table S2: Distribution of MCSS minima of functional groups on the binding surface of AbCR9114, Table S3: Distribution of MCSS minima of functional groups on the binding surface of AbBH151, Table S4: Distribution of MCSS minima of functional groups on the binding surface of Ab4F5.
