# Supplementary material for: Computational Prediction of the Epitopes of HA1 Protein of Influenza Viruses to its Neutralizing Antibodies
Source: Antibodies (Basel). 2018 Dec 20;8(1):2. doi: 10.3390/antib8010002 (PMC6640696; doi:10.3390/antib8010002)
Supplement: Supplementary file 1 [file antibodies-08-00002-s001.pdf]

antibodies

MDPI

Supplementary: Computational Prediction of the Epitopes of HA1 Protein of Influenza Viruses to its Neutralizing Antibodies

Xiaoyan Zeng [1], Fiona S. Legge [2], Chao Huang [1], Xiao Zhang [3], Yongjun Jiao [1], Herbert R. Treutlein [4] and Jun Zeng [2,*]

[1] Institute of Pathogenic Microbiology, Jiangsu Provincial Center for Disease Prevention and Control, Key Laboratory of Enteric Pathogenic Microbiology, Ministry Health, Nanjing 210009, China
[2] MedChemSoft Solutions, Level 3, 2 Brandon Park Drive, Wheelers Hill, VIC 3150, Australia
[3] Key Laboratory of Antibody Technique, Ministry Health, Nanjing 210029, China
[4] Sanoosa Pty Ltd, Level 30, 35 Collins Street, Melbourne, VIC 3000, Australia

* Correspondence: Jun.Zeng@medchemsoft.com; Tel.: +61-413323321

Table S1. Distribution of MCSS minima of functional groups on the binding surface of AbHC19

MCSS	S1	Specific Inter energy kcal/mol	Specific interactions	S2	Specific Inter energy kcal/mol	Specific interactions
BENZ	-	-	-	112	(-10.00, -10.20)	π-π Trp52(H) x 14
IMIA	-	-	-	55	(-10.40, -12.40)	π-π Trp52(H) x 5
PHEN	16	(-10.00, -11.30)	Arg97(H) x 16	107	(-10.00,-16.10)	π-π Trp52(H) x 7
INDO	-	-	-	33	-15.3<br>-15.4	π-π Arg97(H) x 1<br>H bond Tyr107(H) x 1
MAMM	-	-	-	66	-15.60;<br>-15.30,-15.80	Asp98(H) x 1<br>H bond Tyr102(H), Tyr107(H) x1
MGUA	-	-	-	49	-15.40, -16.90, -17.60<br>(-17.29, -18.70)	π-π Arg97(H) x 3<br>H bond Tyr107(H)x 4

Table S2. Distribution of MCSS minima of functional groups on the binding surface of AbCR9114

MCSS	S1	Interaction energy kcal/mol	Specific interactions	S2	Interaction energy kcal/mol	Specific interactions
BENZ	29	(-10.50,-11.1)	π-π Phe49(H)x29	21	(-10.10,-11.40)	π-π Tyr38(L) x 21
PHEN	42	(-10.00,-12.60)	π-π Phe49(H)x13	7	-10.00, -11.30	H bond Asn25(L)x 7
INDO	4	-15.40	π-π Phe49(H)	3	-15.50	H bond Asn25(L)x 1
					-15.20,-15.20	π-π Tyr38(L) x 2
MGUA	-	-	-	4	(-15.00,-17.20)	Asp101(H)
ACET	4	-17.20	Ser46(H) x 1	6	-16.10,-16.10	SER39(L) X 2

Table S3. Distribution of MCSS minima of functional groups on the binding surface of AbBH151

**MCSS**	**B1**	**Interaction energy kcal/mol**	**Specific interactions**	**B2**	**Interaction energy kcal/mol**	**Specific interactions**
BENZ	-	-	-	2	(-10.10, -10.20)	Phe105(H)x2
PHEN	-	-	-	11	(-10.00, -10.40)	Phe105(H)x 5
					(-10.00, -11.70)	Phe105(H),Arg98(H) x [illegible]
ACET	9	up to -16.80	Arg102(H) x 9	30	up to -17.7	Arg102(H) x 17

Table S4. Distribution of MCSS minima of functional groups on the binding surface of Ab4F5

MCSS	B1	Interaction energy kcal/mol	Specific interactions	B2	Interaction energy kcal/mol	Specific interactions	B3	Interaction energy kcal/mol	Specific interactions
BENZ	37	up to -11.00	Leu47(H)x37	6	up to -10.00	Tyr50(H)x6	11	up to -11.20	Tyr33(H), Tyr104(H)x11
IMIA	10	up to -12.90	Leu47(H)x10	3	up to -10.70	Asp118(L)x3	3	up to -10.90	Tyr33(H), Tyr104(H)x3
PHEN	37	up to -13.40	Leu47(H),Tyr50(H)	20	up to -13.80	π-π Tyr104(L), Tyr33(H)	18	up to -13.40	H bonds Asn115(L), Asn32(H)
INDO	11	up to -16.10	H bond Gly101(L)	-			1	up to -10.10	Tyr33(H), Tyr104(H)x3
ACEM	16	up to -11.70	Asp54(L)	4	up to -10.00	Asp51(H)	2	-10.1	Asn32(H)
MAMM	3	(-15.20, -16.00)	Asp54(L) x 3						
MGUA	60	up to -20.8	Asp54(L)						
